# Addressing evidence needs during health crises in the province of Quebec (Canada): a proposed action plan for rapid evidence synthesis

**DOI:** 10.1186/s12913-025-12204-y

**Published:** 2025-01-11

**Authors:** Quan Nha Hong, Esther McSween-Cadieux, Maxime Guillette, Luiza Maria Manceau, Jingjing Li, Vera Granikov, Marie-Pascale Pomey, Marie-Pierre Gagnon, Saliha Ziam, Christian Dagenais, Pierre Dagenais, Alain Lesage, Thomas G. Poder, Martin Drapeau, Valéry Ridde, Julie Lane

**Affiliations:** 1 School of Rehabilitation, Faculty of Medicine, Université de Montréal, C.P. 6128, Succursale Centre-Ville, Montréal (Québec), H3C 3J7 Canada; 2 Centre for Interdisciplinary Research in Rehabilitation of Greater Montreal (CRIR), Montréal, Canada; 3 Department of School and Social Adaptation Studies, Faculty of Education, Université de Sherbrooke, Sherbrooke, Canada; 4 Centre RBC d’expertise universitaire en santé mentale, Université de Sherbrooke, Sherbrooke, Canada; 5 University of Montreal Hospital Research Centre (CRCHUM), Montréal, Canada; 6 Department of Management, Evaluation and Health Policy, School of Public Health, Université de Montréal, Montréal, Canada; 7 Faculty of Nursing, Université Laval, Québec, Canada; 8 School of Business Administration, Université TÉLUQ, Montréal, Canada; 9 Department of Psychology, Faculty of Arts and Sciences, Université de Montréal, Montréal, Canada; 10 Service of Rheumatology, Faculty of Medicine and Health Science, Université de Sherbrooke, Sherbrooke, Canada; 11 Centre de recherche de l’Institut universitaire en santé mentale de Montréal (CR-IUSMM), CIUSSS-de-L’Est-de-L’île-de- Montréal, Montréal, Canada; 12 Department of Educational and Counselling Psychology, Faculty of Education, McGill University, Montréal, Canada; 13 Centre Population et Développement (CEPED), IRD-Université de Paris, Paris, France

**Keywords:** COVID-19, Health crises, Pandemic, Evidence-informed decision-making, Health policymakers, Health managers, Evidence synthesis, Rapid evidence synthesis

## Abstract

**Background:**

The COVID-19 pandemic necessitated the rapid availability of evidence to respond in a timely manner to the needs of practice settings and decision-makers in health and social services. Now that the pandemic is over, it is time to put in place actions to improve the capacity of systems to meet knowledge needs in a situation of crisis. The main objective of this project was thus to develop an action plan for the rapid syntheses of evidence in times of health crisis in Quebec (Canada).

**Methods:**

We conducted a three-phase collaborative research project. First, we carried out a survey with producers and users of rapid evidence syntheses (*n* = 40) and a group interview with three patient partners to prioritize courses of action. In parallel, we performed a systematic mapping of the literature to identify rapid evidence synthesis initiatives developed during the pandemic. The results of these two phases were used in a third phase, in which we organized a deliberative workshop with 26 producers and users of rapid evidence syntheses to identifying strategies to operationalize priorities. The data collected at each phase were compared to identify common courses of action and integrated to develop an action plan.

**Results:**

A total of 14 specific actions structured into four main axes were identified over the three phases. In axis 1, actions on raising awareness of the importance of evidence-informed decision-making among stakeholders in the health and social services network are presented. Axis 2 includes actions to promote optimal collaboration of key stakeholders in the production of rapid evidence synthesis to support decision-making. Actions advocating the use of a variety of rapid evidence synthesis methodologies known to be effective in supporting decision-making are presented in axis 3. Finally, axis 4 is about actions on the use of effective knowledge translation strategies to promote the use of rapid evidence synthesis products to support decision-making.

**Conclusions:**

This project led to the development of a collective action plan aimed at preparing the Quebec ecosystem and other similar jurisdictions to meet knowledge needs more effectively in times of health emergency. The implementation of this plan and its evaluation will enable us to continue to fine-tune it.

**Supplementary Information:**

The online version contains supplementary material available at 10.1186/s12913-025-12204-y.

## Background

The COVID-19 pandemic necessitated the rapid availability of evidence to respond in a timely manner to the needs of practice settings and decision-makers in health and social services [1]. Because of the rapid spread of the virus and the major implications for health and social systems [2], critical decisions had to be made urgently based on data that were often uncertain, contradictory, of variable quality, and rapidly evolving [3–10].

To meet these urgent needs, the teams responsible for evidence synthesis, such as health technology assessment (HTA) agencies, had to quickly adapt their working methods [9]. Evidence syntheses had to be produced faster than ever before, in a matter of weeks, days, or even hours [6, 11]. Thus, the COVID-19 pandemic was linked to a substantial increase in the production of rapid evidence syntheses (RES) to support whenever possible evidence-informed decision-making [12]. RES may be particularly useful under specific conditions (e.g., depending on the urgency of the decision-making, the particular features of the topic, the expectations and needs of users, the availability of resources) [11, 13, 14] or on an interim basis (e.g., pending the production of a systematic review) [13, 15].

In a RES, certain steps in the usual evidence synthesis process are simplified, abbreviated, or omitted to reduce completion time and thereby provide timely information [16, 17]. Accelerating the synthesis process requires several methodological adaptations [9]. Some of the most common include restricting database searches or eligibility criteria (e.g., design type, year of publication, language), using a single person to select studies and extract data, not assessing study quality and risk of bias, and producing a narrative synthesis of the data [9, 13, 18–21]. Generally speaking, the methodological adaptations may have implications for the validity of the conclusions [20, 22, 23]. On one hand, in situations requiring rapid access to evidence, decision-makers would be willing to accept some form of trade-off in terms of validity in exchange for timeliness [9, 15, 24]. On the other hand, maintaining a balance between ensuring the quality of the work, respecting the principles of transparency, and appropriately managing conflicts of interest is a perpetual challenge [25, 26]. Several researchers have, in fact, called for greater transparency to improve the utility, credibility, and reproducibility of RES, as well as their continuous updating [11, 16, 27, 28].

In addition to the methodological adaptations required for evidence production, the studies produced since the start of the pandemic have also generally revealed a lack of synergy and a duplication of synthesis efforts of many teams working on similar topics [26, 29–32]. In 2021, our team conducted a qualitative study to better understand the challenges, the adaptations implemented, and the usefulness of RES during the COVID-19 pandemic from the perspectives of evidence synthesis producers and decision-makers in the province of Quebec (Canada) [26]. Canada has a decentralized, universal health system in which each province is responsible for its own health and social care. Compared with other provinces in Canada, Quebec is the only province that is primarily francophone. Quebec has its own provincial public health institute and HTA agencies as well as several other hospital-based HTA units that produced RES during the pandemic. During the first wave, Quebec was the province that was the most severely hit [33]. The results of our qualitative study highlight several organizational, methodological, professional and personal challenges and adaptations made by teams and organizations during the pandemic [26]. The results of this study helped to identify potential courses of action to improve evidence synthesis processes within and between organizations. These potential courses of action need to be further explored and to draw collective lessons from the COVID-19 pandemic in order to improve the preparedness of organizations involved in evidence synthesis to support decision-making in future health crisis. Also, to manage the uncertainty of the evidence, the study showed that some RES held consultations with experts, health and social services professionals, managers and representatives of professional orders. However, patients and public were rarely involved in RES during the pandemic. This contrasts with a general trend and best practice that favours patients and public involvement in HTA to increase their relevance [34]. There is thus a need to better understand how to involve patients and public in RES in the health crisis context.

It is now an opportune time to put in place what we have learned from the COVID-19 pandemic, and to improve the capacity of systems to meet evidence needs during future health crises. For example, ensuring that evidence synthesis teams should be an integral part of pandemic preparedness plans. The main objective of this research project was to develop an action plan for RES production in times of health crisis. This project was funded by the Québec Covid-Pandemic Network (Fonds de recherche du Québec). This plan is intended to help prepare the Quebec evidence ecosystem to meet evidence needs in times of health emergency, but could also be relevant for other jurisdictions developing systems and processes to manage evidence, particularly in crisis situations.

## Methods

**Fig. 1:**
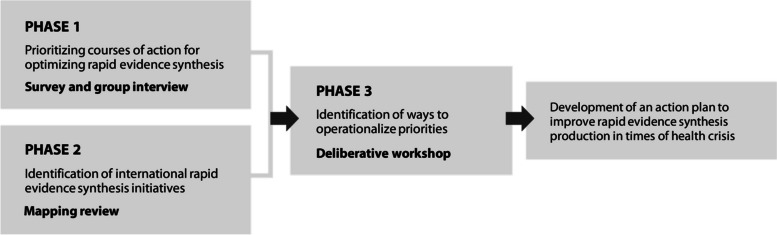
Phases of the project

To develop the action plan, we conducted a three-phase process, with each phase contributing to specifying, refining, and identifying actions deemed as priorities in the context. The first two phases were conducted concurrently, generating outcomes that were subsequently presented and further developed during Phase 3 (Fig. 1). These phases were aimed at: 1) prioritizing courses of action; 2) identifying RES initiatives developed during the pandemic; and 3) identifying ways to operationalize priorities. In this section, we briefly present the methods used in each phase. In the results section, we focus particularly on the overall objective, i.e., the presentation of the action plan, including the supporting data from the different phases (Fig. 1).

We conducted this study in partnership with representatives of various research and practice communities. The study partners were from three provincial institutes in public health, health and social services, six hospital-based HTA agencies, five universities in Quebec, as well as three representatives of the general public, service users, and patient partners. Representatives from a public health agency and a provincial ministry also collaborated on the project. For phases 1 and 3, a monitoring subcommittee was formed to organize data collection, and validate the survey.

### Conceptual framework

We used the Evidence Ecosystem Framework to frame this research project [35]. This framework was used because it clearly presents the different elements and their interrelations in the evidence ecosystems, which was the topic of this project. This framework is composed of four main elements: 1) decision-making by evidence users, such as patients, healthcare providers, and policy makers; 2) production of research by evidence producers such as researchers, graduate students, librarians, and HTA professionals; 3) the engagement between users and producers; and 4) the sociopolitical context within which the evidence is being produced and used [35].

Using this framework and based on the results of our previous study [26], we defined four topics that guided the data collection and analysis: 1) the evidence ecosystem, including actions to strengthen capacities, resources, and formal and informal links among the various system players involved in data production and use; 2) knowledge translation for decision-making by users, including actions to ensure that RES are adapted to target audiences and easily accessible, in a timely manner, to encourage their use; 3) evidence synthesis methodology for research production, including actions aimed at defining, specifying, adapting, and sharing methodological practices for producing RES, with a view to reconciling rigour and speed; and 4) the involvement of partners, including actions to determine ways of consulting or involving partners (e.g., patients, service users) in RES, to facilitate the engagement between users and producers.

### Data collection

#### Phase 1—Online survey and group interview

The aim of this phase was to prioritize courses of action for optimizing RES. To reach this aim, we conducted a survey that was developed based on the results of our qualitative study conducted in 2021 [26]. The questionnaire was validated with the subcommittee members. It consisted of three main parts. The first asked four questions about the profile of participants (e.g., types of organizations, experiences with RES). In the second part, 21 courses of action were proposed to promote the production of RES in times of health crisis. Participants were asked to rate their level of importance and feasibility on a 5-point scale (not at all, not much, relatively, very or extremely important) and suggest other courses of action as needed in an open-ended question. In the third part, two questions were asked to explore whether they had collaborated with patient partners to produce a RES during the pandemic and to invite them to share their ideas on how to better consider their perspectives when producing RES.

A snowball strategy was used to recruit participants. Invitation email was sent to the project partners inviting them to complete the survey and to forward the invitation within their team or organization. The survey was also posted on social media such as LinkedIn. Interested parties had access to the survey via the LimeSurvey platform. The estimated time to complete the survey was approximately 15 to 20 min. Responses were collected between February 7 and March 7, 2023. A total of 40 people completed the survey. The participants worked in different centers, with some affiliated to more than one centers: health and social services centres (*n* = 17), provincial institutes (*n* = 11), ministries (*n* = 3), research centers (*n* = 5), universities (*n* = 6), and non-profit organisations (*n* = 1). Six mentioned other organisations (e.g., self-employed).

In addition, we held a group interview with three patient partners. These individuals were referred by the Centre of Excellence on Partnership with Patients and the Public (CEPPP). The group interview took place on February 22, 2023, and lasted two hours. The goal was to capture their experiences in the production of a RES related to COVID-19 and identify avenues for improvement. Two researchers (EMC, QNH) participated in the group (facilitator and note taker), and the questions were focused on the projects in which the individuals had participated, the successes in terms of their involvement, the facilitating conditions, and the challenges encountered when participating as patient partners in evidence synthesis.

We descriptively analyzed the quantitative survey data collected in phase 1. For the qualitative data collected in the open-ended questions of the surveys and the group interview, we performed a content analysis [36].

#### Phase 2—Mapping review

We performed a systematic mapping review of the literature to identify RES initiatives put in place during the pandemic COVID-19. A mapping review aims to collate, describe and catalogue existing evidence on a specific topic [37]. Publications dated between January 1st, 2020 and October 14, 2022, were searched in three databases (Medline (OVID), Embase (OVID), CINAHL (EBSCO)). The search strategy was developed by a specialized librarian and checked by another reviewer using the PRESS checklist [38]. The search strategy included a combination of free text and controlled vocabulary on the concepts of COVID-19 and evidence synthesis, with limits on year (2020 onward) and language (English or French) (for full search strategies, see Additional file 2). All the records were transferred to Covidence, an online software for managing systematic reviews. In addition, four other sources were consulted: 1) Google search with the following terms: COVID rapid evidence synthesis, COVID rapid review methods, and COVID rapid evidence response service (first 10 pages consulted); 2) websites of organizations involved in evidence synthesis—list drawn from the International Network of Agencies for Health Technology Assessment (INAHTA) and the Guidelines International Network (GIN); 3) backward citation tracking on included papers; and 4) articles on selected initiatives identified on websites or Google Scholar.

Two independent reviewers were involved in the selection of papers. All publications that focused on evidence synthesis initiatives put in place in response to the COVID-19 pandemic were considered for inclusion. More specifically, evidence synthesis initiatives could consist of services (e.g., rapid review services) and/or tools (e.g., literature hub, quality appraisal tool). Papers that provided guidance for future evidence synthesis initiatives and lessons learned were also included. There was no restriction on the types of papers published or countries. Papers were excluded if they reported results of rapid reviews on a specific COVID-19 related topic or were methodological reviews.

The data extracted were: year, country, name of the initiative, description of the initiative, organization that developed or offered the initiative, challenges encountered, and lessons learned. Two reviewers independently extracted data from five papers to pretest the data extraction form. Afterwards, one reviewer extracted data from the remaining papers and a second reviewer validated the data extracted.

For the data extracted from the literature, we tabulated them in an Excel file to map the characteristics of each evidence synthesis initiative. Then, we grouped the initiatives based on their type (service or tool) and purpose. In addition, we classified the tools based on the evidence synthesis step for which they were developed (i.e., search, selection, extraction, appraisal, synthesis). A total of 85 initiatives were identified (for flow diagram and list of initiatives, see Additional file 2).

#### Phase 3—Deliberative workshop

In March 2023, a virtual half-day deliberative workshop [39] was held to identify ways to operationalize the priority courses of action.

A snowball sampling [40] was used to recruit participants who had participated in RES activities during the pandemic. An email was sent to the project partners inviting them to attend the workshop and to share the invitation with key people in their organization. In addition, a workshop registration link was included at the end of the survey (Phase 1) so that interested parties could register. Notices were also posted on social media. A total of 26 people attended the workshop, including 17 from teams producing RES to support decision-making in health and social services organizations in Quebec, four from the research community, two from a ministry, and three patient partners and citizens.

The results of the consultation (Phase 1) and the initiatives mapping (Phase 2) served as the basis for discussions at the workshop. The main results of each phase were sent to the participants in advance by email. During the workshop, following the presentation of the preliminary results of the first two phases, participants engaged in a first round of breakout deliberations on two themes (evidence ecosystem and knowledge translation). The participants could choose the breakout room they prefer to join. These first subgroup sessions, which lasted 45 min, were followed by 30 min of collective feedback and discussion to improve the proposals put forward on each theme. A second round of breakout deliberations was conducted for two other themes (evidence synthesis methodology and involvement of partners), as well as a large group feedback session to obtain the perspectives and suggestions of all participants. The workshop was recorded, transcribed and the data were analysed using a content analysis to identify the strategies to operationalize the courses of action [36].

The process of integrating the results from the three phases into an action plan involved three main steps. First, the findings from each phase were synthesized and translated into potential courses of action, which were categorized according to predefined thematic areas to ensure coherence with the conceptual framework. The detailed results of each phase are available in additional files 1 to 3. Second, the potential actions were compared across phases, with priority given to those that emerged frequently, were rated as highly important, or were further operationalized and refined during the deliberative workshop. This iterative analysis allowed to identify and prioritize actions based on their relevance, feasibility, and potential impact. Finally, the prioritized actions were consolidated into a comprehensive draft action plan developed by the project’s three principal investigators (JL, EMS, QNH). The draft action plan was reviewed by project’s partners to ensure its applicability and contextual relevance.

## Results

**Fig. 2:**
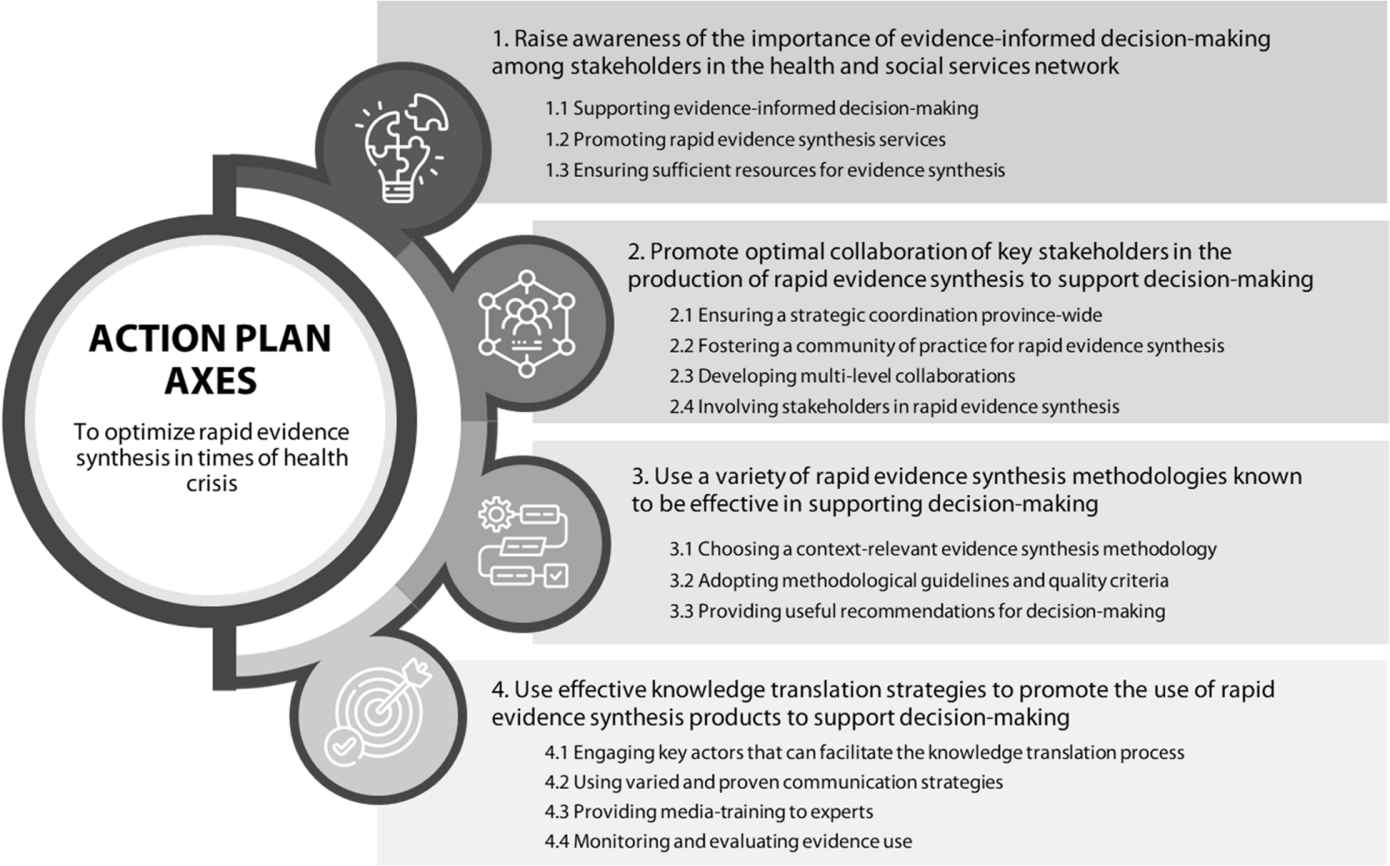
Action plan summary

This results section focuses on the collective action plan that is the culmination of the elements that emerged from the three phases of the project. These elements have been grouped around four main axes that structure the action plan. These axes are presented, as well as the 14 more specific actions proposed to support these axes. Figure 2 provides a summary of the action plan.

## AXIS 1 – Raise awareness of the importance of evidence-informed decision-making among stakeholders in the health and social services network

### Supporting evidence-informed decision-making

Promoting an organizational culture in the health and social services network that values evidence-based decision-making was the course of action considered most important in the survey. Almost 93% of participants considered this course to be very or extremely important. Proposals on how to operationalize this approach included implementing communication strategies to stress the importance of evidence-informed decisions and raising awareness among users of the various evidence syntheses. To achieve this, the proposal was to make them more aware of the range of types of synthesis methods and to explain that the choice of method depends on the nature of the decision-making needs and of the context. At the workshop, it was stressed that RES should not become the norm in all contexts. During the times of crisis given the urgent need for the response, more methodological shortcuts might be acceptable. Participants in the deliberative workshop also stressed the importance, in reports, of being transparent about methodological trade-offs and their impacts so as to better inform decision-making. They also noted the need to sensitize decision-makers to the importance of independence and impartiality in the production of syntheses.

### Promoting RES services

This action is about setting up teams in organizations that would be dedicated to coordinating or producing RES in times of emergency and to make the services of teams and organizations that support decision-makers in the health and social services system and the general public more widely known. This course of action was recommended by participants in the open-ended questions of the survey and discussed during the workshop.

In addition, in the initiatives found in the literature, it is possible to identify many teams that were set up to produce different products for evidence syntheses (*n* = 47). Four broad categories of products can be identified: 1) literature monitoring, to keep abreast of the latest scientific evidence and track progress in developments and research trends; 2) summaries of scientific evidence, which allow the results of studies or syntheses to be made available in a short, popularized format; (3) rapid evidence synthesis, to integrate research findings from studies into the body of evidence available on a topic; various synthesized products were developed, such as rapid responses, rapid guidelines, clinical guidelines, and practice points; and the methods used for the syntheses were variable; and finally, 4) the continuous updating of rapid evidence syntheses to take into account the rapid evolution of scientific evidence. The terms “living” or “rolling” are used to characterize these products.

### Ensuring sufficient resources for evidence synthesis

It is important to ensure that decision-support teams and organizations have the means and resources needed to carry out their mission, especially in times of crisis, and that their mission be considered essential. During the workshop, it was noted that several people with tasks related to evidence synthesis (HTA professionals, information specialists) were relieved from their usual duties during the pandemic in Quebec, when they could have contributed to supporting decision-making in a time of crisis.

Given the short time allotted for producing syntheses, one facilitating measure mentioned in the literature is to enlist a team with expertise in evidence syntheses, with members who have previously worked together [11, 32, 41–45]. There is a need for staff who are trained and dedicated, have established relationships of trust, and are able to work in a variety of contexts. Moreover, due to the magnitude of the task during the pandemic, some authors also cautioned that the workload was excessive and unsustainable over the long term. They called for a reduction in the pace of work to avoid staff burnout [46, 47] and for investment in infrastructure to enable rapid deployment of highly qualified staff [48].

## AXIS 2 – Promote optimal collaboration of key stakeholders in the production of RES to support decision-making

### Ensuring strategic coordination province-wide

To ensure strategic coordination province-wide in the event of a health crisis, participants in the survey and the deliberative workshop recommended implementing a "crisis cell". This infrastructure would involve having a strategic committee attached to the Ministry of Health and Social Services to quickly identify evidence needs and bring together key expertise, including managers from the ministerial departments concerned (e.g., public health administration, clinical departments), managers of decision-making teams and organizations, and the Ministry of Public Security. Such coordination would also involve a tactical committee that would bring together the decision-support teams to pool resources.

Several infrastructures were quickly set up to meet urgent demands for scientific evidence during the COVID-19 pandemic. To ensure the sustainability of the structures put in place, several authors have stressed the importance of institutional commitment, adequate funding, an integrated organizational structure, a formal definition of each person’s role, and qualified personnel [49–52]. The same observations have also been made with respect to the technological tools developed that require a long-term financial investment in order to ensure that web platforms are updated and to make them accessible and useful for user communities [53].

In addition, ensuring that rapid synthesis processes can meet the needs of all regions of Quebec was considered the fourth most important course of action in the survey. As such, a provincial coordination infrastructure is also needed to ensure that local contextual and experiential data from the regions are taken into account.

### Fostering a community of practice for RES

In line with the previous course of action, participants suggested strengthening the interactions among decision-support teams and organizations in order to pool efforts, promote mutual learning, and share methods and the projects they are working on in times of emergency. This would involve creating a virtual sharing platform where the teams producing evidence syntheses would share their monitoring findings, submit their productions, and announce the synthesis projects underway to better coordinate productions. The importance of strengthening collaboration within the network (both intra- and inter-organization) was suggested by both survey respondents and workshop participants, as it remains a major challenge.

Moreover, improving the sharing of RES to avoid duplication was an important course of action in the survey, as 90% of participants considered it very or extremely important. Also, during the workshop several strategies for improving evidence centralization in times of crisis were proposed, such as pooling information monitoring or grouping products within a single strategic location.

### Developing multi-level collaborations

This course of action is aimed at encouraging and strengthening provincial, national, and international collaborations in the event of a health crisis in order to improve concerted action. Also, the importance of creating more links with research teams and engaging university researchers in decision-making processes was raised in the survey and at the workshop.

Given the global scale of the COVID-19 pandemic, many organizations in each country have been mobilized to identify, summarize, assess, and synthesize existing evidence to inform decision-making. Various efforts have been made to create networks and consortia among the evidence-producing communities to foster inter-organizational collaboration. In this context, it is essential to foster collaboration between organizations at provincial, national, and international levels [32, 42, 44, 48, 54, 55]. This is necessary to avoid duplication and overlapping of work, to prioritize issues and spread out the projects to be done, and to share methodologies and resources available and under development [6, 11, 32, 42, 56]. For example, the COVID-19 Evidence Network to support Decision-making (COVID-END) was an international network initiated at McMaster University that brought together more than 50 organizations around the world with expertise in evidence synthesis, HTA, and the development of guidelines to support decision-making [54]. COVID-END offered a variety of services and resources for the production of evidence syntheses.

### Involving stakeholders in RES

It is recommended to involve key people from the beginning of a RES project. These may include stakeholders, health professionals, members of the public, service users or patient partners, and researchers. Several strategies to operationalize this course of action were proposed, such as recognizing in times of crisis the importance of different types of evidence (including experiential and contextual evidence) in syntheses, maintaining communication with partners throughout the project, encouraging interdisciplinary engagement, and having a librarian involved from the beginning. Involving stakeholders in RES was considered less important in the survey compared to other actions (see Additional file 1) but was widely discussed during the workshop.

With respect to involving patient partners, users, and members of the public in RES in times of crisis, several strategies were proposed during the group interview and the workshop: valuing their expertise; calling on networks already mobilized or existing communities; involving partners promptly to keep them mobilized and responsive; valuing diverse profiles and promoting representativeness; including the partners throughout the process by considering them to be full members; jointly defining expectations and providing guidelines; addressing disability situations and discussing possible accommodations; and providing compensation modalities tailored to their situation.

The literature also emphasizes the need for greater involvement of stakeholders (e.g., service users, clinicians, or managers) from the beginning to the end of the RES process [44, 48, 54, 57, 58]. This is suggested to promote better consideration of the factors that could influence the conclusions and contextualization of the results, as well as to formulate feasible and acceptable recommendations. A mechanism for feedback and communication with stakeholders should be set up from the start of the process [59]. For example, one initiative identified was created by the Cochrane Collaboration, which set up a group of stakeholders experienced in partnership engagement. The purpose of this group was to recruit people at risk of or with COVID-19, connect them with production settings, and provide guidance and resources to train synthesis producers and service users on the evidence synthesis process and partnership engagement (*Cochrane’s COVID-19 consumer rapid response task group*).

## AXIS 3 – Use a variety of RES methodologies known to be effective in supporting decision-making

### Choosing a context-relevant evidence synthesis methodology

In times of health crisis, it has been proposed during the workshop that the focus should be on a proportionality of efforts approach. This is a strategy whose aim is to determine the right methodology to address a decision-making need based on the context and nature of the request. It has been said that more biases could be acceptable when a response to the need cannot wait. This would allow for a decompartmentalized continuum of methodologies (beyond the traditional rapid review and systematic review) and the use of a range of evidence synthesis methods. To properly determine methodological choices in RES, the stage of clarifying the request from managers and decision-makers is retained as a priority, even when the request is urgent. This stage is done to specify and validate their needs as well as to sequence the steps involved in producing the synthesis, in order to respond to the request as quickly as possible.

### Adopting methodological guidelines and quality criteria

It is important to apply the recognized methodological benchmarks and quality criteria for each type of method used. These criteria must uphold integrity, transparency, rigour, and ethics. The importance of transparency and of further clarifying the limitations of the methodological adaptations and, above all, their impact on decision-making needs was repeated many times during the workshop. In this regard, it was recommended that it be made clear in the reports which findings come from preprint studies and non-peer-reviewed reports, among others. Independent double verification was an adaptation often made during the COVID-19 pandemic to speed up evidence synthesis processes. However, it was recommended that a certain way of working in pairs be consistently applied to limit potential biases despite the urgency to act.

In the literature, it is suggested that methodological guides be developed to standardize and formalize the RES process [32, 41, 48, 60–62]. During the pandemic, several organizations published methodological guides on RES, such as Cochrane, the Haute Autorité de Santé (HAS), the Institut national d’excellence en santé et en services sociaux (INESSS), and the National Institute for Health and Care Excellence (NICE). This process needs to be clear, rigorous, and transparent, but also flexible and adaptable due to the evolving nature of the literature.

### Providing useful recommendations for decision-making

The importance of improving practices to make clear, specific, and operational recommendations, where applicable, was another important course of action in the survey, with 92.5% of participants rating it as very or extremely important. During the workshop, it was noted that when a RES is received at the ministry, work must be done to take its conclusions and translate them into decisions according to different scenarios. The roles and responsibilities of the evidence synthesis communities in translating data into recommendations were discussed several times. Even though this is what decision-makers need, many producers expressed their discomfort in making recommendations, because in a crisis context, the synthesis processes do not comply with the usual quality standards. Some preferred using the terms “main findings” or “positions” in their RES rather than issuing recommendations. It was suggested during the workshop that consideration be given to separating the recommendations from the evidence synthesis document or providing more contextual information before making recommendations. In anticipation of the next health crisis, it was suggested to have guidelines for integrating experiential, contextual and scientific evidence into clear, specific and operational recommendations, with a view to better supporting decision-making.

## AXIS 4—Use effective knowledge translation strategies to promote the use of RES products to support decision-making

### Engaging key actors that can facilitate the knowledge translation process

It was recommended that people with expertise in communication and knowledge translation be involved from the outset of the project in order to develop a dissemination plan. During the workshop, the suggestion was made to identify individuals who would help facilitate exchanges between stakeholders. These intermediaries can play a translation role between the decision-makers’ needs and the decision-support teams producing the evidence syntheses, enhancing the feasibility of implementation through ongoing communication and iterative feedback. Key persons in these roles should be located at the Ministry of Health and Social Services to support ministerial actors in integrating evidence into the decision-making processes with the help of evidence synthesis products.

Along these lines, it was pointed out during the workshop that one of the keys to success during the pandemic was the close collaboration established with potential users from the beginning of the rapid syntheses. These collaborations facilitated the triangulation of information essential to decision-making (experiential, contextual, and clinical-administrative evidence), made it possible to assess the feasibility of the recommendations, and improved community adherence to recommendations. People in this intermediary role helped create the networks needed for knowledge translation.

To expedite the dissemination of results, the literature also recommends collaborating with the knowledge translation team from the beginning of the project and maintaining regular communications [11, 55, 57]. This will make it possible to prepare a knowledge translation plan early in the process and to develop appropriate strategies that can be deployed quickly to reach user communities and support the effective application of evidence syntheses outputs.

### Using varied and proven communication strategies

The aim of this course of action is to encourage the use of diverse communication strategies known to be effective in reaching different audiences (e.g., offering incentives to encourage people to participate in activities, writing executive summaries in plain language with key messages highlighted, ensuring the accessibility of documents, using existing communication platforms to reach target audiences [26]). In the survey, 87.5% of participants considered it important or extremely important to present the RES in a brief, easy-to-navigate, and visually appealing format. To this end, recommendations were made to take into consideration the literacy level of the target population, to adopt an inclusive writing angle, and to consider innovative strategies for reaching some populations that are more resistant to certain recommendations (e.g., collaborating with champions or leaders recognized by these populations). Also, during the workshop, participants stressed the importance of providing more training in knowledge translation and strategic writing to the teams producing rapid syntheses, as RES need to get to the essentials, clearly and concisely. Workshop participants also raised the importance of translating scientific knowledge into practical information for settings to facilitate its uptake in times of crisis (e.g., developing guides for practice settings).

To facilitate timely access to results, the literature suggests diversifying knowledge translation strategies, such as the use of email, social media, YouTube, blogs, and podcasts [11, 63]. The use of simple, succinct summaries that highlight key messages for user communities is also recommended [11]. Finally, products can be easily accessed via web platforms, such as directories [48, 49].

### Providing media-training to experts

The recommendation was made that people who are asked to participate in media outings be supported and trained to use accessible language and to be aware of the posture they should adopt (e.g., communicating scientific facts and not an opinion). In the survey, 85% of participants deemed improving the communication of RES when they are disseminated in the media and social networks to be very or extremely important. Also, in the workshop, the importance of reviewing how public communications were conducted during the COVID-19 pandemic was raised. Explaining more clearly what is being done to deal with a situation filled with uncertainty and the fact that the situation is evolving in line with the science would allow to readjust the aim and correct the messages in step with this evolution. Transparency was recommended, as well as taking into account the impacts of such transparency on the population. This would help the public better understand the processes and what the decisions are based on. In this respect, on several occasions, participants stressed the importance of generally strengthening the capacity of organizations to take into account literacy levels, in order to adapt communications to different target audiences. In addition, when organizations’ positions diverged from those of other organizations or provinces, it was suggested that the reasons for the differences be clearly explained.

### Monitoring and evaluating evidence use

It would be important to monitor the use of evidence products and fund research projects to assess the impacts of these products on decision-making processes in times of health crisis. For example, if certain recommendations are not implemented by decision-makers, practice settings, or the general population, such monitoring and evaluation processes would be useful to improve and readjust the communication and knowledge translation strategies. To do this, collaborations could be undertaken between decision-support teams, organizations, and research teams to evaluate the products’ impacts on decision-makers, health professionals, or the general public, and thus ensure better monitoring. This is in line with a recommendation highlighted at the workshop of further engaging academic researchers.

In the literature, a few studies on the evaluation of the use of RES products were identified [48, 64, 65]. For example, a mixed methods study was conducted with healthcare practitioners to assess the impact of guidelines produced by the Australian National COVID-19 Clinical Evidence Taskforce. Data were collected on guideline awareness, relevance, ease of use, trustworthiness, value, importance of updating, use, strengths, and opportunities for improvement. Results showed that more than 50% of respondents reported having used the guidelines for various purposes, such as to inform treatment decisions, compare with other guidelines, and seek new evidence [65]. In other studies, qualitative interviews with key informants were conducted to explore how and to what extent RES products were useful to stakeholders [48, 64].

## Discussion

The aim of this study was to develop collectively an action plan for producing RES in times of health crisis. The three phases of data collection led to the development of a plan with four main axes and 14 courses of action aimed at better preparing the health system for future emergency situations. In addition to supporting the development of this plan, this study uncovered several findings that warrant discussion related to the use of science in decision-making, collaborations within the ecosystem and pooling of efforts, technological advances for evidence synthesis, the importance of common methodological guidelines, knowledge translation, and the involvement of users.

Before the pandemic, evidence-informed decision-making was already recognized as crucial and in need of improvement by many international organizations. In 2024, the Global Commission of Evidence, led by McMaster University, reiterated the need for every jurisdiction to formalize and strengthen systems to contextualize evidence to inform decision-making [66]. The participants in this study shared the same concern, as evidenced by the very high priority given to this course of action. The COVID-19 pandemic has given decision-makers a better understanding of the value of knowing the state of the science and how to access the latest scientific evidence. This is a potentially favourable context for making advances in the use of science, and thus a unique opportunity to be seized by those in charge of international organizations, policy-makers, and managers. On one hand, decision-makers and managers need to be even more aware of the scientific culture, and on the other, public health experts and researchers need to better understand the political environment in which decisions are made and adapt their communication accordingly [67]. We should keep in mind that several resources exist and can be useful in promoting such reciprocal awareness [21, 68–71].

The present study also shows the importance of strengthening collaboration mechanisms and developing strong networks of actors in the ecosystem to deal with the next health emergency. This is also a concern reported in the literature, namely the need to optimize coordination and sharing among the various evidence-producing communities [31, 60, 66, 72]. As such, this concern appears to be widely shared beyond the Quebec context.

In Quebec, it was proposed that such a course of action takes the form of pooled or centralized efforts at the provincial level to promote strategic coordination of the pandemic response. An interesting initiative documented in the province of Saskatchewan (Canada) was the establishment of a single electronic platform to receive questions from decision-makers, managers, and clinicians, prioritize them, and provide responses based on the best evidence available at the time by the COVID-19 Evidence Support Team [49]. Besides facilitating evidence circulation, the platform allowed for more uniform and consistent decision-making across the province, initiating a learning health system and facilitate communication and coordination among the various stakeholders [49]. It may be useful to explore what form such a dedicated platform could take in Quebec and other jurisdictions.

The mapping review identified a large number of technological initiatives developed during the pandemic. In particular, it identified more than 20 tools to facilitate and accelerate literature retrieval (e.g., directories, surveillance systems). The mapping also identified initiatives based on advanced technology such as machine learning (e.g., automated systems) that were promising. Those were mainly used for document retrieval, and less for the other steps of synthesis. In addition, various promising initiatives were developed but unknown to the production teams. For example, MetaInsight [73] and MetaCOVID [74] can perform network meta-analyses using data that have already been extracted and evaluated. These initiatives greatly speed up the process, as the selection, extraction, and evaluation have already been done and the synthesis is automatically generated based on the desired parameters.

The technological developments found in the mapping review were not discussed in the workshop. This can represent a significant gap between research and practice, as research teams are developing many technological resources and tools to improve evidence synthesis processes, but these seem to be little, or not at all, known or used by organizations producing syntheses and would certainly warrant exploration to assess their potential. Workshop participants also stressed that academic researchers could be more mobilized. In the event of another pandemic, such initiatives could be developed more quickly, and closer collaborations between researchers and production organizations and teams would be needed to make these initiatives better known and more useable. Thus, to bridge this gap, it would appear important to: 1) develop such technological tools in collaboration with practice settings, such as organizations producing syntheses to support decision-making; 2) strengthen collaboration between research teams and synthesis-producing organizations in carrying out syntheses; and 3) create accessible technologies that can be easily used in practice and limit the duplication of similar tools.

The COVID-19 pandemic has necessitated numerous adaptations of evidence synthesis products. The mobilization of RES in this specific type of context needs to be enhanced, and at the same time, minimum methodological benchmarks should be formulated to guarantee a certain threshold of quality in the work produced. The literature amply shows the need for guidelines to better define the adaptations that can be made to maintain a balance between speed and rigour, and to identify those that pose too great a risk of compromising quality [41, 59]. The risks of bias induced by methodological adaptations are unavoidable, although it is possible to try to mitigate them and explain them clearly with greater transparency [32]. Rehfuess et al. [55] go even further by suggesting that it would be necessary to distinguish between the intended standards for RES and those produced in emergency mode (e.g., in less than a day), as was the case during the COVID-19 pandemic. Such guidelines would prove invaluable in better guiding the work of decision-support teams.

The importance of presenting RES products in a short, easy-to-navigate, and visually appealing format was a concern raised during the study. This very much underscores the fact that clear and effective knowledge translation is more important than ever in times of crisis, when decision-making and practice communities are overwhelmed with information and have little time to take in evidence. Indeed, short summaries (such as a two-pager [75] or a policy brief [76]) can promote the use of data when key messages are highlighted from the start for users and clear, simple language is used [7, 11, 63, 77, 78].

Users' involvement can also be useful in increasing the relevance, accessibility, applicability, and credibility of syntheses products [11, 48, 57, 58, 79]. Despite this, many participants in this study considered it to be generally unfeasible in a pandemic context. This appears consistent with other findings that indicate users involvement was relatively low during the pandemic [59, 79]. There could be several reasons for this, including lack of access to networks of partners already mobilized and responsive, or lack of guidance on how and when to involve partners meaningfully in RES when timelines are short in times of emergency. A field of reflection could be set up to better define how to mobilize users and patient partners in a health crisis situation. Garritty et al. [79] list various contributions users can make at all stages of the synthesis (pre-planning, planning and initiation, production, and post-synthesis), as well as key elements to consider when involving partners. Such an article could provide food for thought on the high-priority involvement modalities best suited to a health crisis context.

### Study limitations

Some of this study’s limitations could stem from the snowball recruitment approach, which might have introduced certain biases, in that people less close to the partners’ networks might have been less likely to be solicited to participate in the data collection. Moreover, since most of the project partners were in organizations that produce evidence syntheses, recruitment by these actors may partly explain why few decision-makers participated in the surveys. Also, the number of participants was small, which limit the representativeness of the target population. With respect to the literature search, it is likely that the number of initiatives identified is an underestimate, as the search strategy was limited to initiatives in English and French and published between January 2020 and October 2022. As for the deliberative workshop, the participants might have influenced each other and tended to conform to ideas expressed by others. Also, some people take up more space in discussions, while others may be less comfortable speaking in groups. The facilitators nevertheless made sure to create a climate conducive to interactions and made every effort to engage everyone's point of view. Moreover, the priority courses of action and the strategies identified to operationalize them appear to echo the various needs and issues identified in the international scientific literature. Nonetheless, each country and jurisdiction has its own characteristics and challenges, including those inherent in existing infrastructures, types of health systems, and cultural dimensions.

## Conclusions

This project led to the development of a collective action plan aimed at preparing the Quebec ecosystem to meet evidence needs more effectively in times of health emergency, and could inspire other jurisdictions interested by these topics. This plan was shared with stakeholders from the Quebec Ministry of Health and Social Services. Further work is needed to refine, implement and test the action plan in preparation for a future health crisis. Moreover, work could also be carried out to make the action plan applicable to other countries and jurisdictions, in particular by adapting the actions and methods.

## Supplementary Information

Supplementary Material 1.

## Data Availability

The datasets generated and analyzed during the current study are not publicly available due to integrity of participants but are available from the corresponding author on reasonable request.
