# Supplementary material for: Addressing evidence needs during health crises in the province of Quebec (Canada): a proposed action plan for rapid evidence synthesis
Source: BMC Health Serv Res. 2025 Jan 11;25:61. doi: 10.1186/s12913-025-12204-y (PMC11725205; doi:10.1186/s12913-025-12204-y)
Supplement: Supplementary file 1 — Supplementary Material 1. [file 12913_2025_12204_MOESM1_ESM.docx]

**Additional files**

Additional file 1: Results of phase 1

Additional file 2: Database search strategy and results of phase 2

Additional file 3: Results of phase 3

# Additional file 1. Results of phase 1

The purpose of the survey was to assess the perceived importance and feasibility of 21 actions to improve rapid evidence synthesis during public health emergencies. 40 participants completed the survey. In addition, 3 patients partners participated in a group interview.

|  |  | 1. Not at all | 2. A little | 3. Moderately | 4. Very | 5. Extremely | Don’t know | MEAN |
| --- | --- | --- | --- | --- | --- | --- | --- | --- |
| 1. Promote an organizational culture that values evidence-informed decision-making | IMPORTANT | 0 (0%) | 0 (0%) | 2 (5%) (5%) | 7 (17.5%) | 30 (75%) | 1 (2.5%) | 4.72 |
|  | FEASIBLE | 0 (0%) | 2 (5%) | 11 (27.5%) | 11 (27.5%) | 13 (32.5%) | 3 (7.5%) | 3.95 |
| 2. Improve the sharing of rapid evidence synthesis results by teams and organizations to avoid duplication and optimize resources. | IMPORTANT | 0 (0%) | 0 (0%) | 3 (7.5%) | 6 (15%) | 30 (75%) | 1 (2.5%) | 4.69 |
|  | FEASIBLE | 0 (0%) | 3 (7.5%) | 11 (27.5%) | 14 (35%) | 10 (25%) | 2 (5%) | 3.81 |
| 3. Enhance practices for formulating clear, precise, and operational recommendations when applicable. | IMPORTANT | 0 (0%) | 0 (0%) | 1 (2.5%) | 11 (27.5%) | 26 (65%) | 2 (5%) | 4.66 |
|  | FEASIBLE | 0 (0%) | 1 (2.5%) | 10 (25%) | 15 (37.5%) | 14 (35%) | 0 (0%) | 4.05 |
| 4. Ensure that the existing rapid evidence synthesis production processes meet the needs of stakeholders from all regions of Quebec. | IMPORTANT | 0 (0%) | 1 (2.5%) | 3 (7.5%) | 8 (20%) | 28 (70%) | 0 (0%) | 4.58 |
|  | FEASIBLE | 2 (5%) | 8 (20%) | 12 (30%) | 11 (27.5%) | 5 (12.5%) | 2 (5%) | 3.24 |
| 5. Present rapid evidence syntheses in a concise, user-friendly, and visually appealing format. | IMPORTANT | 0 (0%) | 0 (0%) | 4 (10%) | 9 (22.5%) | 26 (65%) | 1 (2.5%) | 4.56 |
|  | FEASIBLE | 0 (0%) | 0 (0%) | 8 (20%) | 10 (25%) | 21 (52.5%) | 1 (2.5%) | 4.33 |
| 6. Identify the methodological aspects to minimally describe in reports to enhance transparency. | IMPORTANT | 0 (0%) | 0 (0%) | 2 (5%) | 15 (37.5%) | 21 (52.5%) | 2 (5%) | 4.50 |
|  | FEASIBLE | 0 (0%) | 0 (0%) | 2 (5%) | 16 (40%) | 20 (50%) | 2 (5%) | 4.47 |
| 7. Increase the capacity for timely production of rapid evidence syntheses (e.g., resources and qualified personnel). | IMPORTANT | 0 (0%) | 2 (5%) | 1 (2.5%) | 14 (35%) | 22 (55%) | 1 (2.5%) | 4.44 |
|  | FEASIBLE | 1 (2.5%) | 7 (17.5%) | 14 (35%) | 12 (30%) | 2 (5%) | 4 (10%) | 3.19 |
| 8. Improve communication of products in media and social networks to promote understanding. | IMPORTANT | 1 (2.5%) | 1 (2.5%) | 2 (5%) | 11 (27.5%) | 23 (57.5%) | 2 (5%) | 4.42 |
|  | FEASIBLE | 0 (0%) | 0 (0%) | 12 (30%) | 8 (20%) | 13 (32.5%) | 7 (17.5%) | 4.03 |
| 9. Share practices and methodological guidelines of rapid evidence syntheses among teams and organizations. | IMPORTANT | 0 (0%) | 0 (0%) | 4 (10%) | 15 (37.5%) | 20 (50%) | 1 (2.5%) | 4.41 |
|  | FEASIBLE | 1 (2.5%) | 3 (7.5%) | 7 (17.5%) | 16 (40%) | 13 (32.5%) | 0 (0%) | 3.93 |
| 10. Clarify rapid evidence synthesis methodologies based on questions, needs, context, and resources. | IMPORTANT | 0 (0%) | 0 (0%) | 6 (15%) | 12 (30%) | 22 (55%) | 0 (0%) | 4.40 |
|  | FEASIBLE | 0 (0%) | 2 (5%) | 12 (30%) | 12 (30%) | 14 (35%) | 0 (0%) | 3.95 |
| 11. Strengthen connections between various entities producing rapid evidence syntheses in Quebec. | IMPORTANT | 0 (0%) | 1 (2.5%) | 5 (12.5%) | 11 (27.5%) | 22 (55%) | 1 (2.5%) | 4.38 |
|  | FEASIBLE | 1 (2.5%) | 1 (2.5%) | 16 (40%) | 13 (32.5%) | 6 (15%) | 3 (7.5%) | 3.59 |
| 12. Improve the process of framing the assessment question and the decision-making need with the requester. | IMPORTANT | 0 (0%) | 1 (2.5%) | 6 (15%) | 11 (27.5%) | 22 (55%) | 0 (0%) | 4.35 |
|  | FEASIBLE | 1 (2.5%) | 2 (5%) | 10 (25%) | 14 (35%) | 11 (27.5%) | 2 (5%) | 3.84 |
| 13. Combine the written document with knowledge translation activities through verbal communication with requesters, to ensure greater appropriation of the results. | IMPORTANT | 0 (0%) | 0 (0%) | 6 (15%) | 15 (37.5%) | 19 (47.5%) | 0 (0%) | 4.33 |
|  | FEASIBLE | 0 (0%) | 3 (7.5%) | 11 (27.5%) | 8 (20%) | 17 (42.5%) | 1 (2.5%) | 4.00 |
| 14. Improve mechanisms to track completed or ongoing rapid evidence syntheses. | IMPORTANT | 0 (0%) | 1 (2.5%) | 6 (15%) | 13 (32.5%) | 20 (50%) | 0 (0%) | 4.30 |
|  | FEASIBLE | 0 (0%) | 2 (5%) | 11 (27.5%) | 19 (47.5%) | 6 (15%) | 2 (5%) | 3.76 |
| 15. Establish and sustain monitoring mechanisms to facilitate continuous updating of rapid evidence syntheses. | IMPORTANT | 0 (0%) | 1 (2.5%) | 6 (15%) | 14 (35%) | 19 (47.5%) | 0 (0%) | 4.28 |
|  | FEASIBLE | 0 (0%) | 4 (10%) | 14 (35%) | 11 (27.5%) | 11 (27.5%) | 0 (0%) | 3.73 |
| 16. Enhance mechanisms for prioritizing requests for rapid evidence synthesis within organizations. | IMPORTANT | 0 (0%) | 0 (0%) | 7 (17.5%) | 16 (40%) | 17 (42.5%) | 0 (0%) | 4.25 |
|  | FEASIBLE | 0 (0%) | 2 (5%) | 14 (35%) | 14 (35%) | 8 (20%) | 2 (5%) | 3.74 |
| 17. Share results with the requester as soon as possible rather than waiting for the written document dissemination, thus reducing delays. | IMPORTANT | 0 (0%) | 1 (2.5%) | 9 (22.5%) | 9 (22.5%) | 20 (50%) | 1 (2.5%) | 4.23 |
|  | FEASIBLE | 1 (2.5%) | 3 (7.5%) | 4 (10%) | 13 (32.5%) | 17 (42.5%) | 2 (5%) | 4.11 |
| 18. Raise awareness of the processes that are used by different teams and organizations to produce rapid evidence syntheses. | IMPORTANT | 0 (0%) | 1 (2.5%) | 9 (22.5%) | 12 (30%) | 18 (45%) | 0 (0%) | 4.18 |
|  | FEASIBLE | 0 (0%) | 1 (2.5%) | 11 (27.5%) | 17 (42.5%) | 10 (25%) | 1 (2.5%) | 3.92 |
| 19. Strengthen exchanges between producers and requesters of rapid evidence syntheses during the project. | IMPORTANT | 1 (2.5%) | 0 (0%) | 7 (17.5%) | 14 (35%) | 17 (42.5%) | 1 (2.5%) | 4.18 |
|  | FEASIBLE | 0 (0%) | 4 (10%) | 10 (25%) | 12 (30%) | 12 (30%) | 2 (5%) | 3.84 |
| 20. Determine how to better integrate patient, service-user and citizen knowledge in rapid synthesis. | IMPORTANT | 1 (2.5%) | 0 (0%) | 8 (20%) | 12 (30%) | 17 (42.5%) | 2 (5%) | 4.16 |
|  | FEASIBLE | 2 (5%) | 2 (5%) | 15 (37.5%) | 11 (27.5%) | 5 (12.5%) | 5 (12.5%) | 3.43 |
| 21. Involve citizens, service users and patient more closely in the production of rapid evidence synthesis. | IMPORTANT | 1 (2.5%) | 4 (10%) | 10 (25%) | 6 (15%) | 16 (40%) | 3 (7.5%) | 3.86 |
|  | FEASIBLE | 4 (10%) | 8 (20%) | 12 (30%) | 5 (12.5%) | 5 (12.5%) | 6 (15%) | 2.96 |

**Other actions suggested by survey participants (open-ended questions) and a group interview.**

- Define what would be considered scientific data in "times of crisis.

- Take into consideration the local contextual and experiential data from Quebec's regions.

- Adapt mechanisms for capturing the needs of different requesters (e.g., government, hospital).

- Increase collaborations with research teams.

- Create a rapid evidence synthesis team within organizations.

- Create a community of practice (e.g., to help track completed and ongoing syntheses).

- Centralize knowledge to make it easier to find evidence syntheses produced in Quebec.

- Establish a "crisis unit" to facilitate internal and external communication.

- Identify appropriate tools to disseminate rapid evidence syntheses to stakeholders.

- Develop a communication plan for products with knowledge translation specialists.

- Disseminate in plain language, regardless of subject or format.

- Ensure that documents are easily accessible on the platforms people consult.

- Involve synthesis producers in implementing recommendations.

- Enhance the value of all types of evidence to prevent results from being biased and limited in their application.

- Describe the methodology used to collect experiential evidence.

- Clarify the value of rapid evidence synthesis in relation to other HTA projects.

- Develop a collaborative approach between those requesting and producing the evidence syntheses.

- Clarify the roles and responsibilities of the requester versus the producer.

- Promote cultural change to recognize and value the expertise of patients, service users and citizens.

- Involve patients, service users and citizens from the beginning of the project and define expectations together.

- Involve patients, service users and citizens throughout the project and treat them as full members of the project.

- Openly discuss disability situations with patients, service users and citizens and discuss potential accommodations.

- Provide compensation modalities adapted to the situation of patients, service users and citizens.

- Facilitate access to patients, service users and citizens who are initiated, trained and able to mobilize quickly.

- Use already mobilized networks or existing communities of patients, service users and citizens.

- Value diverse patient, service user and citizen profiles and promote community representativeness.

- Provide guidance and feedback to patients, service users and citizens on the work carried out.

- Ensure that patients, service users and citizens feel listened to, valued, and respected.

# Supplementary file 2. Database search strategy and results of phase 2

The purpose of the literature mapping was to identifying rapid evidence synthesis initiatives implemented internationally during COVID-19.

**Database search strategy (2022-10-14)**

| **Databases** | **Search strategy** |
| --- | --- |
| Medline (Ovid) | 1. COVID-19/or exp COVID-19 Testing/ or COVID-19 Vaccines/ or SARS-CoV-2/  2. (nCoV* or 2019nCoV or 19nCoV or COVID19* or COVID or SARS-COV-2 or SARSCOV-2 or SARS-COV2 or SARSCOV2 or SARS coronavirus 2 or Coronavirus disease 2019).ab,kf,kw,ti.  3. (Evidence adj1 (synthes* or service* or review*)).ab,kf,kw,ti.  4. (Rapid adj1 (review* or synthes* or response* or evidence)).ab,kf,kw,ti.  5. “Knowledge synthes*”.ab,kf,kw,ti.  6. 1 or 2  7. 3 or 4 or 5  8. 6 and 7  9. limit 8 to (yr="2020 -Current" and (english or french)) |
| Embase (Ovid) | 1. coronavirus disease 2019/  2. COVID-19 testing/  3. SARS-CoV-2 vaccine/  4. Severe acute respiratory syndrome coronavirus 2/  5. (nCoV* or 2019nCoV or 19nCoV or COVID19* or COVID or SARS-COV-2 or SARSCOV-2 or SARS-COV2 or SARSCOV2 or SARS coronavirus 2 or Coronavirus disease 2019).ab,kf,kw,ti.  6. (Evidence adj1 (synthes* or service* or review*)).ab,kf,kw,ti.  7. (Rapid adj1 (review* or synthes* or response* or evidence)).ab,kf,kw,ti.  8. "Knowledge synthes*".ab,kf,kw,ti.  9. 1 or 2 or 3 or 4 or 5  10. 6 or 7 or 8  11. 9 and 10  12. limit 11 to ((english or french) and yr="2020 -Current") |
| CINAHL (Ebsco) | S1. (MM “COVID-19”) OR (MM “COVID-19 Testing”) OR (MM “COVID-19 Vaccines”) OR (MM “COVID-19 Pandemic”) OR (MM “SARS-CoV-2”)  S2 . TI ( nCoV* or 2019nCoV or 19nCoV or COVID19* or COVID or SARS-COV-2 or SARSCOV-2 or SARS-COV2 or SARSCOV2 or SARS coronavirus 2 or Coronavirus disease 2019 ) OR AB ( nCoV* or 2019nCoV or 19nCoV or COVID19* or COVID or SARS-COV-2 or SARSCOV-2 or SARS-COV2 or SARSCOV2 or SARS coronavirus 2 or Coronavirus disease 2019 )  S3. TI ( (Evidence N1 (synthes* or service* or review*)) ) OR AB ( (Evidence N1 (synthes* or service* or review*)) )  S4. TI ( (Rapid N1 (review* or synthes* or response* or evidence)) ) OR AB ( (Rapid N1 (review* or synthes* or response* or evidence)) )  S5. TI "Knowledge synthes*" OR AB "Knowledge synthes*"  S6. S1 OR S2  S7. S3 OR S4 OR S5  S8. S6 AND S7 Limiters - Published Date: 20200101-; Language: English, French |

**Flow diagram**

**
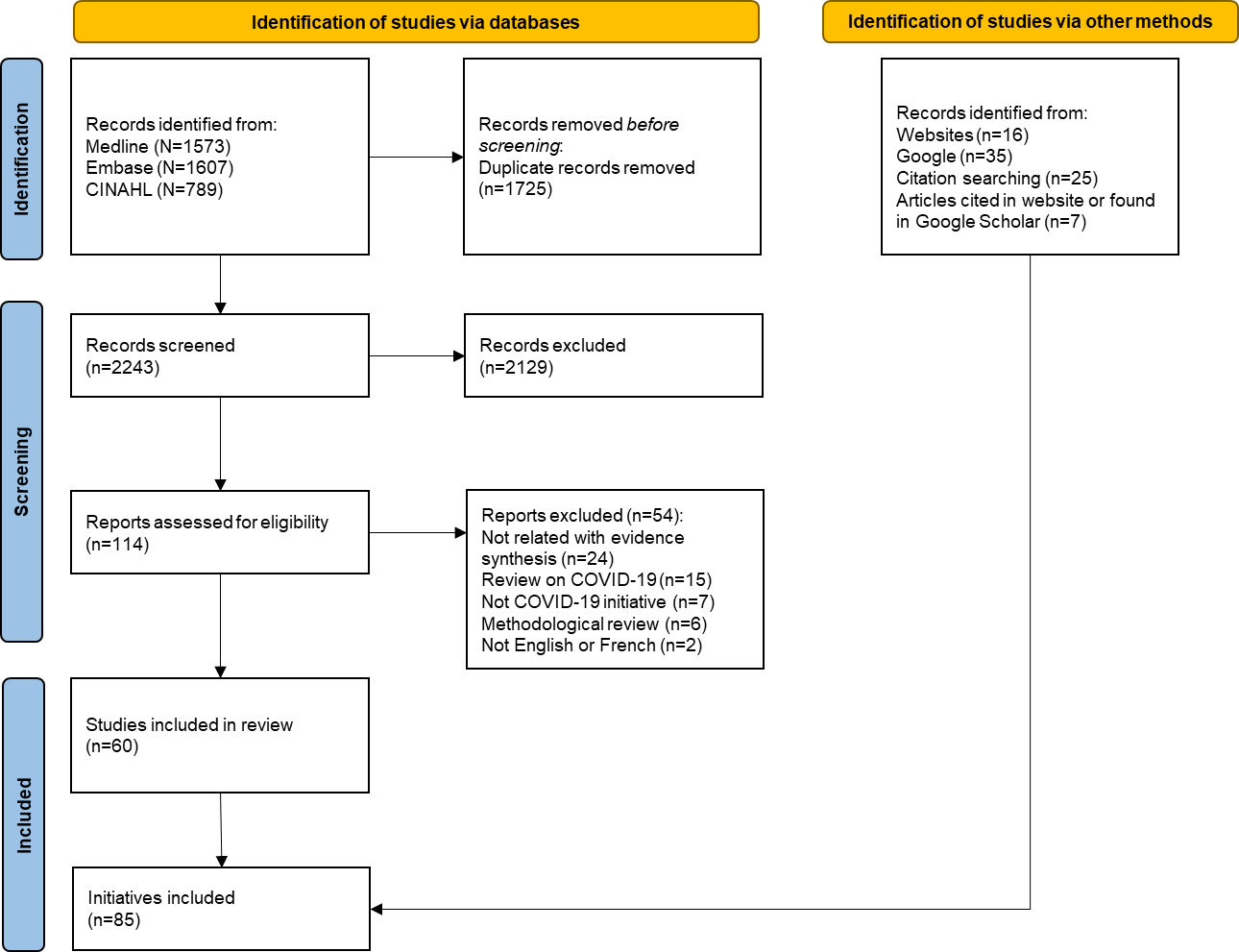
**

**List of initiatives identified in the literature mapping (n=85)**

| **Initiatives** | **Countries** | **Organisations** | **References** |
| --- | --- | --- | --- |
| ACP living practice points | USA | American College of Physicians (ACP) | [1] |
| [Australian National COVID-19 Clinical Evidence Taskforce](https://clinicalevidence.net.au/covid-19/) | Australia | Consortium of 32 organisations in health | [2–5] |
| [BIP4COVID19](https://www.kaggle.com/datasets/mathurinache/bip4covid19) | Greece | Institute for the Management of Information Systems (IMSI) | [6] |
| [CADTH COVID-19 Evidence Portal](https://www.cadth.ca/cadth-covid-19-evidence-portal) | Canada | Canadian Agency for Drugs and Technologies in Health (CADTH) |  |
| [CanCOVID](https://cancovid.ca/) | Canada | CanCOVID |  |
| Children's Colorado Rapid Evidence Analysis and Dissemination System (CCREADS) | USA | Children's Hospital Colorado (CHCO) (COVID-19 Scientific Advisory Council (SAC)) | [7] |
| CSN COVID-19 Rapid Response Team (CCRRT) | Canada | Canadian Society of Nephrology (CSN) | [8] |
| [Cochrane COVID-19 Study Register (CCSR)](https://covid-19.cochrane.org/) | International | Cochrane | [9] |
| [Cochrane COVID Rapid Reviews](https://www.cochranelibrary.com/covid-19) | International | Cochrane | [10] |
| [Cochrane Rehabilitation REH-COVER action](https://rehabilitation.cochrane.org/covid-19/reh-cover-interactive-living-evidence) | International | Cochrane Rehabilitation | [11,12] |
| COVID-19 Knowledge Extraction framework (COKE project) | Italy | Italian National Council of Research (CNR) et University of Bologna | [13] |
| [CORD-19: COVID-19 Open Research Dataset](https://allenai.org/data/cord-19) | USA | Allen Institute for Artificial Intelligence |  |
| [COVID-19: a living systematic map of the evidence](https://eppi.ioe.ac.uk/cms/Projects/DepartmentofHealthandSocialCare/Publishedreviews/COVID-19Livingsystematicmapoftheevidence/tabid/3765/Default.aspx) | England | EPPI-Center |  |
| [COVID-19 Best Evidence Front Door](https://frontdoor.knack.com/covidbestevidence) | USA | University of Michigan |  |
| [COVID-19 Clinical Guidance Cell](https://www.sign.ac.uk/covid-19-guidance-developed-in-partnership/) | England | Scottish Intercollegiate Guidelines Network (SIGN) |  |
| [COVID-19 Consumer rapid response group](https://consumers.cochrane.org/covid-19-consumer-rapid-response-group-resources) resources | International | Cochrane |  |
| [Covid-19 Critical Intelligence Unit (CIU)](https://aci.health.nsw.gov.au/covid-19/critical-intelligence-unit) | Australia | NSW Health Agency for Clinical Innovation | [14] |
| [COVID-19 Evidence Network to support Decision-making (COVID-END)](https://www.mcmasterforum.org/networks/covid-end) | International | COVID-END | [15,16] |
| [COVID-Evidence](https://covid-evidence.org/) | Switzerland and USA | University of Basel, Meta-Research Innovation Center at Stanford |  |
| [COVID-19 Primer](https://covid19-science.primer.ai/5322aaf6-974b-46da-92e0-ac0a0f754a21) | USA | Primer.ai |  |
| [COVID-19 Evidence Alerts from McMaster PLUS](https://plus.mcmaster.ca/COVID-19/About) | Canada | Health Information Research Unit (HiRU) à McMaster University |  |
| [COVID-19 Evidence ecosystem (CEOsys)](https://covid-evidenz.de/) | Germany | CEOsys | [9,17] |
| [COVID-19 Evidence review webiste](https://www.covid19reviews.org/) | USA | Veterans Affairs Evidence Synthesis Program (VA ESP) | [18] |
| [COVID-19 Evidence Support Team (CEST)](https://saskhealthauthority.libguides.com/covid-19/repository/home) | Canada | Saskatchewan Health Authority, College of Medicine at the University of Saskatchewan (USASK), Health Quality Council, Ministry of Health | [19,20] |
| [COVID-19 Evidence synthesis from SPOR](https://sporevidencealliance.ca/key-activities/covid-19-evidence-synthesis/) | Canada | SPOR Evidence Alliance |  |
| [COVID-19 Evidence Synthesis Team](https://www.hiqa.ie/areas-we-work/health-technology-assessment/covid-19-publications) | Ireland | Health Information and Quality Authority (HIQA) | [21–24] |
| [COVID-19 HTA Response Team](https://past.htai.org/hta-support-for-covid-19/covid-19-hta-response-team/) | International | Health Technology Assessment International (HTAi) |  |
| COVID-19 integrated Knowledge Translation (iKT) Unit | Canada | Vancouver Coastal Health (VCH) Authority | [25] |
| [COVID-19 Knowledge Accelerator (COKA)](https://gps.health/covid-19-knowledge-accelerator-coka/) | USA | EBSCO Health Innovations, EBSCO Information Services | [26] |
| COVID Knowledge Graph | Germany | Fraunhofer Institute for Algorithms and Scientific Computing (SCAI), University of Bonn | [27] |
| COVID-19 knowledge synthesis and discovery framework | Canada | NICHE Research Group, Faculty of Computer Science, Dalhousie University | [28,29] |
| COVID-19 literature surveillance | Canada | Public Health Agency of Canada | [30] |
| [COVID-NMA Project](https://covid-nma.com/) | International | COVID-NMA Consortium | [31] |
| [COVID-19 L.OVE repository](https://app.iloveevidence.com/covid19) | Chili | Epistemonikos Foundation | [32] |
| [COVID Quest](https://crowd.cochrane.org/) | International | Cochrane | [9,33] |
| [COVID-19 Rapid Evidence Reviews Group (CORRE) - ISARIC](https://isaric.org/research/covid-19-clinical-research-resources/covid-19-rapid-evidence-reviews-group-corre/) | International | International Severe Acute Respiratory and emerging Infection Consortium (ISARIC) |  |
| [Covid-19 search filter](https://app.2dsearch.com/query) | England | University of Nottingham, University of London | [34] |
| [COVIDscholar](https://covidscholar.org/) | USA | Berkeley | [35] |
| [CTS COVID rapid clinical guidelines](https://cts-sct.ca/guideline-library/) | Canada | Canadian Thoracic Society (CTS) | [36] |
| [Latest evidence on COVID-19](https://www.ecdc.europa.eu/en/covid-19) | Europe | European Centre for Disease Prevention and Control |  |
| [eCOVID RecMap](https://covid19.recmap.org/) | Canada | McMaster University, Cochrane Canada and 10 other institutions worldwide |  |
| EMERGE Tool | India | Netaji Subhash Chandra Bose Government Medical College | [37,38] |
| [Emergency Evidence Response Service (EERS)](https://evidencesynthesisireland.ie/covid-19/) | Ireland | Evidence Synthesis Ireland, Cochrane Ireland, HRB Trials Methodology Research Network |  |
| EUnetHTA COVID-19-response | Europe | European Network for Health Technology Assessment (EUnetHTA) | [39,40] |
| [Evidence Aid - Coronavirus (COVID-19)](https://evidenceaid.org/evidence/coronavirus-covid-19/?language=en) | England | Evidence Aid |  |
| [Evidence Collaboration on COVID-19 (ECC-19)](https://sites.google.com/view/ecc19/home) | International | Coordination by WHO |  |
| [Evidence synthesis response to COVID-19 crisis - 3ie](https://www.3ieimpact.org/about-us/Evidence-synthesis-response-COVID-19-crisis) | International | International Initiative for Impact Evaluation (3ie) |  |
| [Evidence synthesis unit](https://www.health.gov.on.ca/en/pro/ministry/research/evidence_synthesis.aspx) | Canada | Ministry of Health, Ministry of Long-term Care of Ontario, CADTH, Centre for effective practice (CEP), Cochrane Canada, Dalla Lana School of public health, Institute for Clinical Evaluative Sciences, McMaster Health Forum/RISE, Ministry of Health, OH Cancer Care Ontario, Ontario Health Quality, Ontario Hospital Association, Ontario Medical Association, Public Health Ontario, Rapid Evidence Access Link, SPOR Evidence Alliance, Trillium Health Partners |  |
| FOAMED community | England | Manchester University NHS Foundation Trust, University of Manchester, Salford Royal NHS Foundation Trust, Lancaster University, Stockport NHS Foundation Trust, Lancashire Teaching Hospitals NHS Foundation Trust, Royal College of Emergency Medicine, Manchester Metropolitan University | [41] |
| Framework for evidence synthesis programs | USA | Mayo Clinic | [42] |
| [Guidelines International Network COVID-19 Taskforce](https://g-i-n.net/wp-content/uploads/2021/05/20201001-GIN-COVID-19-Taskforce-Position-Paper-Navigating-evidence-guidance-in-a-pandemic.pdf) | International | Guidelines International Network (GIN) | [43] |
| [HAS réponses rapides](https://www.has-sante.fr/jcms/p_3168771/fr/methode-d-elaboration-des-reponses-rapides-dans-le-cadre-du-covid-19) | France | Haute autorité de santé (HAS) |  |
| [HSS/ Horizon Scanning for Covid19](https://aihta.at/page/hss-horizon-scanning-fuer-covid19/en) | Austria | Austrian Institute for Health Technology Assessment (AIHTA) |  |
| [INESSS – Réponses rapides](https://www.inesss.qc.ca/covid-19.html) | Canada | Institut national d’excellence en santé et en services sociaux (INESSS) |  |
| International Task Force Network of Coronavirus Disease 2019 (InterNetCOVID-19) | International | Cochrane Croatia, Cochrane Sweden, Cochrane Brazil, Cochrane US Network, Cochrane Iran | [44] |
| Irish COVID-19 Evidence for General Practitioners | Ireland | IRISH College of General Practitioners (ICGP), Association of University Departments of General Practice in Ireland (AUDGPI), HRB Primary Care Clinical Trials Network in Ireland (PCCTNI) | [23] |
| [iSearch COVID- 19 Portfolio](https://icite.od.nih.gov/covid19/search/) | USA | National Institutes of Health | [45] |
| [LitCOVID](https://www.ncbi.nlm.nih.gov/research/coronavirus/) | USA | National Institute of Health | [46] |
| LIVING Project | International | Copenhagen University Hospital, Lund University, University of Ioannina, Li Ka Shing Knowledge Institute, McMaster University, Hamilton, University of Southern Denmark | [47–49] |
| [Map – Meta evidence](https://meta-evidence.co.uk/wp-content/uploads/2020/03/280320-1.html) | England | Campbell UK & Ireland |  |
| [MetaInsigth COVID-19](https://crsu.shinyapps.io/metainsightcovid/) | England | Complex Reviews Support Unit (University of Glasgow, University of Leicester, London School of Hygiene and Tropical Medicine) | [50] |
| [MetaCOVID](https://covid-nma.com/metacovid/) | France | COVID-NMA, Université Paris Cité, Inserm, Cochrane France | [51] |
| [NCCMT Covid-19 Rapid Evidence Service](https://www.nccmt.ca/fr/rapid-evidence-service) | Canada | National Collaborating Centre for Methods and Tools (NCCMT) | [52–54] |
| [Neo-CLEAR project](https://docs.google.com/spreadsheets/d/1L9tsrLn9a7LMql_nnUfMA3uS1SSurrj4XUh2yT2bEUc/edit#gid=1867332198) | USA | University of Colorado | [55] |
| [NICE programme of rapid guidelines](https://www.nice.org.uk/process/pmg20/resources/developing-nice-guidelines-the-manual-appendices-2549710189/chapter/appendix-l-interim-process-and-methods-for-guidelines-developed-in-response-to-health-and-social) | England | National Institute for Health and Care Excellence (NICE) | [56] |
| [NIPH Rapid review team](https://www.fhi.no/en/sys/news/?blockId=90733&ownerPage=45271&language=en) | Norway | Norwegian Institute of Public Health (NIPH) | [57] |
| [Ontario COVID-19 Science Advisory Table](https://covid19-sciencetable.ca/about/) | Canada | Public Health Ontario (PHO) |  |
| [Oxford COVID-19 Evidence Service](https://www.cebm.net/oxford-covid-19-evidence-service/) | England | Centre for Evidence-Based Medicine |  |
| [PROSPERO fast-track registration of protocols related to COVID-19](https://www.crd.york.ac.uk/prospero/) | England | PROSPERO |  |
| Rapid Advice Guidelines (RAG) | China | National Clinical Research Center for Child Health and Disorders | [58] |
| RCOG COVID-19 guidance development | England | Royal College of Obstetricians and Gynaecologist (RCOG) | [59] |
| [REAL COVID-19 Rapid evidence access link](https://www.covid19real.ca/) | Canada | Dalla Lana, University of Toronto |  |
| [Reboot: COVID-Cancer Project](https://rebootrx.org/covid-cancer) | USA | Reboot Rx | [60] |
| [rEM (rapid evidence map)](https://www.sciome.com/rem/) | USA | Sciome LLC, Research Triangle Park | [61] |
| Research Integrity Assessment tool | Germany | University Hospital Wuerzburg | [62] |
| [Scientific Advisory Group COVID-19 Recommendations](https://www.albertahealthservices.ca/assets/info/ppih/if-ppih-covid-19-sag-rapid-review-methodology.pdf) | Canada | Alberta Health Services |  |
| [Semi-Automated Rapid Review Workflow](https://www.kaggle.com/code/jasonnance/semi-automated-sr-covid-19-hypercoagulable-state/notebook) | USA | RTI Center for Data Science |  |
| South African National Essential Medicines - COVID-19 review team | South Africa | National Department of Health of South Africa | [63] |
| [Synopsis of COVID-19 Key Research Articles](https://www.publichealthontario.ca/en/diseases-and-conditions/infectious-diseases/respiratory-diseases/novel-coronavirus/articles) | Canada | Public Health Ontario (PHO) |  |
| Thai National critical care allocation guideline | Thailand | Health Intervention and Technology Assessment Program (HITAP), International Health Policy Program (IHPP) | [64] |
| [UKHSA COVID-19 Rapid Evidence Service](https://ukhsalibrary.koha-ptfs.co.uk/covid19rapidreviews/#Rapid) | England | UK Health Security Agency (UKHSA) |  |
| [UNCOVER (Usher Network for COVID-19 Evidence Reviews) registry](https://www.ed.ac.uk/usher/uncover/register-of-reviews) | England | University of Edinburgh | [65] |
| TRICE (Template for Rapid Iterative Consensus of Experts) | England | British Psychological Society's COVID-19 Behavioural Science and Disease Prevention Taskforce | [66] |
| [Wales COVID-19 Evidence Centre](https://healthandcareresearchwales.org/about-research-community/wales-covid-19-evidence-centre) | England | Health and Care Research Wales |  |
| [WHO COVID-19 Research Database](https://www.who.int/emergencies/diseases/novel-coronavirus-2019/global-research-on-novel-coronavirus-2019-ncov) | International | World Health Organization (WHO) |  |

**References**

1. Qaseem A, Yost J, Forciea MA, Jokela JA, Miller MC, Obley A, et al. The development of living, rapid practice points: Summary of methods from the scientific medical policy committee of the american college of physicians. Ann Intern Med. 2021;174(8):1126‑32.

2. Bell RJ. Evidence synthesis in the time of COVID-19. Climacteric. 2021;24(3):211‑3.

3. Millard T, Elliott JH, Green S, Tendal B, Vogel JP, Norris S, et al. Awareness, value and use of the Australian living guidelines for the clinical care of people with COVID-19: an impact evaluation. J Clin Epidemiol. 2022;143:11‑21.

4. Tendal B, Vogel JP, McDonald S, Norris S, Cumpston M, White H, et al. Weekly updates of national living evidence-based guidelines: methods for the Australian living guidelines for care of people with COVID-19. J Clin Epidemiol. 1 mars 2021;131:11‑21.

5. Turner T, Elliott J, Tendal B, Vogel JP, Norris S, Tate R, et al. The Australian living guidelines for the clinical care of people with COVID-19: What worked, what didn’t and why, a mixed methods process evaluation. PLoS ONE. 2022;17(1 January):e0261479.

6. Vergoulis T, Kanellos I, Chatzopoulos S, Pla Karidi D, Dalamagas T. BIP4COVID19: Releasing impact measures for articles relevant to COVID-19. Quant Sci Stud. 1 déc 2021;2(4):1447‑65.

7. Rao S, Kwan BM, Curtis DJ, Swanson A, Bakel LA, Bajaj L, et al. Implementation of a Rapid Evidence Assessment Infrastructure during the Coronavirus Disease 2019 (COVID-19) Pandemic to Develop Policies, Clinical Pathways, Stimulate Academic Research, and Create Educational Opportunities. J Pediatr. 2021;230:4-8.e2.

8. Nesrallah G, Gilmour L, Levin A, Mustafa R, Soroka S, Zimmerman D. The CSN COVID-19 Rapid Response Program. Can J Kidney Health Dis [Internet]. 2020;7. Disponible sur: http://www.cjkhd.org/

9. Metzendorf MI, Featherstone RM. Evaluation of the comprehensiveness, accuracy and currency of the Cochrane COVID-19 Study Register for supporting rapid evidence synthesis production. Res Synth Methods. 2021;12(5):607‑17.

10. Bero LA. Producing Independent, Systematic Review Evidence: Cochrane’s Response to COVID-19. Am J Public Health. 2020;110(7):952‑3.

11. Arienti C, Kiekens C, Bettinsoli R, Engkasan JP, Frischknecht R, Gimigliano F, et al. Cochrane Rehabilitation: 2020 annual report. Eur J Phys Rehabil Med. 2021;57(2):303‑8.

12. Negrini S, Ceravolo MG, Cote P, Arienti C. A systematic review that is « “rapid” » and «  “living” »: A specific answer to the COVID-19 pandemic. J Clin Epidemiol. 2021;138:194‑8.

13. Golinelli D, Nuzzolese AG, Sanmarchi F, Bulla L, Mongiovì M, Gangemi A, et al. Semi-Automatic Systematic Literature Reviews and Information Extraction of COVID-19 Scientific Evidence: Description and Preliminary Results of the COKE Project. Information. 28 févr 2022;13(3):117.

14. Levesque JF, Sutherland K, Watson D, Currow DC, Bolevich Z, Koff E. Learning systems in times of crisis: The Covid-19 Critical Intelligence Unit in New South Wales, Australia. NEJW Catal. 2020;1(6):1‑11.

15. Dewidar O, Kawala BA, Antequera A, Tricco AC, Tovey D, Straus S, et al. Methodological guidance for incorporating equity when informing rapid-policy and guideline development. J Clin Epidemiol. 2022;150:142‑53.

16. McCaul M, Tovey D, Young T, Welch V, Dewidar O, Goetghebeur M, et al. Resources supporting trustworthy, rapid and equitable evidence synthesis and guideline development: results from the COVID-19 evidence network to support decision-making (COVID-END). J Clin Epidemiol. 2022;151:88‑95.

17. Rehfuess EA, Burns JB, Pfadenhauer LM, Krishnaratne S, Littlecott H, Meerpohl JJ, et al. Lessons learnt: Undertaking rapid reviews on public health and social measures during a global pandemic. Res Synth Methods. 2022;13(5):558‑72.

18. Vela K. COVID-19 Evidence Reviews website: a VA effort to catalog and curate COVID-19 evidence reviews. J Med Libr Assoc JMLA. 2022;110(1):109‑12.

19. Groot G, Baer S, Badea A, Dalidowicz M, Yasinian M, Ali A, et al. Developing a rapid evidence response to COVID-19: The collaborative approach of Saskatchewan, Canada. Learn Health Syst. 2022;6(1):e10280.

20. Groot G, Witham S, Badea A, Baer S, Dalidowicz M, Reeder B, et al. Evaluating a learning health system initiative: Lessons learned during COVID‐19 in Saskatchewan, Canada. Learn Health Syst [Internet]. 9 oct 2022 [cité 20 mai 2023]; Disponible sur: https://onlinelibrary.wiley.com/doi/10.1002/lrh2.10350

21. Sharp MK, Forde Z, McGeown C, O’Murchu E, Smith SM, Ryan M, et al. Irish Media Coverage of COVID-19 Evidence-Based Research Reports From One National Agency. Int J Health Policy Manag. 2021;12.

22. Ryan M. Evidence Synthesis to support the public health response during the COVID-19 pandemic...14th European Public Health Conference (Virtual), Public health futures in a changing world, November 10-12, 2021. Eur J Public Health. 2021;31:iii244‑5.

23. Clyne B, Hynes L, Kirwan C, McGeehan M, Byrne P, Killilea M, et al. Perspectives on the production, and use, of rapid evidence in decision making during the COVID-19 pandemic: a qualitative study. BMJ Evid-Based Med [Internet]. 30 juin 2022 [cité 10 sept 2022]; Disponible sur: https://ebm.bmj.com/content/early/2022/06/30/bmjebm-2021-111905

24. Clyne B, Walsh KA, O’Murchu E, Sharp MK, Comber L, K.K OB, et al. Using preprints in evidence synthesis: Commentary on experience during the COVID-19 pandemic. J Clin Epidemiol. 2021;138:203‑10.

25. Dix-Cooper L, Dawes M, Park M. Vancouver Coastal Health informed COVID-19 response by applying rapid review methodology: reply to Tricco. J Clin Epidemiol. 2021;134:167‑71.

26. Alper BS, Richardson JE, Lehmann HP, Subbian V. It is time for computable evidence synthesis: The COVID-19 Knowledge Accelerator initiative. J Am Med Inform Assoc. 2020;27(8):1338‑9.

27. Domingo-Fernández D, Baksi S, Schultz B, Gadiya Y, Karki R, Raschka T, et al. COVID-19 Knowledge Graph: a computable, multi-modal, cause-and-effect knowledge model of COVID-19 pathophysiology. Bioinformatics. 2021;37(9):1332‑4.

28. Barrett M, Abidi SSR, Daowd A, Abidi S. A Knowledge Graph of Mechanistic Associations Between COVID-19, Diabetes Mellitus, and Chronic Kidney Disease. Stud Health Technol Inform. 2022;290:304‑8.

29. Barrett M, Daowd A, Abidi SSR, Abidi S. A Knowledge Graph of Mechanistic Associations Between COVID-19, Diabetes Mellitus and Kidney Diseases. Stud Health Technol Inform. 2021;281:392‑6.

30. Corrin T, Ayache D, Baumeister A, Young K, Pussegoda K, Ahmad R, et al. COVID-19 literature surveillance--A framework to manage the literature and support evidence-based decision-making on a rapidly evolving public health topic. Can Commun Dis Rep. 2023;49(1).

31. Boutron I, Chaimani A, Meerpohl JJ, Hróbjartsson A, Devane D, Rada G, et al. The COVID-NMA Project: Building an evidence ecosystem for the COVID-19 pandemic. Ann Intern Med. 15 déc 2020;173(12):1015‑7.

32. Verdugo-Paiva F, Vergara C, Ávila C, Castro-Guevara JA, Cid J, Contreras V, et al. COVID-19 Living Overview of Evidence repository is highly comprehensive and can be used as a single source for COVID-19 studies. J Clin Epidemiol. 2022;195‑202.

33. Noel-Storr A, Gartlehner G, Dooley G, Persad E, Nussbaumer-Streit B. Crowdsourcing the identification of studies for COVID-19-related Cochrane Rapid Reviews. Res Synth Methods. 2022;13(5):585‑94.

34. Shokraneh F, Russell-Rose T. Lessons from COVID-19 to future evidence synthesis efforts: first living search strategy and out of date scientific publishing and indexing industry. J Clin Epidemiol. 2020;123:171‑3.

35. Dagdelen J, Trewartha A, Huo H, Fei Y, He T, Cruse K, et al. COVIDScholar: An automated COVID-19 research aggregation and analysis platform. PLOS ONE. 18(2):e0281147.

36. Gupta S. Providing clinical guidance in the middle of a global pandemic: Caveats and opportunities. Can J Respir Crit Care Sleep Med. 2021;5(6):343‑5.

37. Yadav SK, Agrawal V, Agarwal P, Sharma D. Rapid Scoping Review of Laparoscopic Surgery Guidelines During the COVID-19 Pandemic and Appraisal Using a Simple Quality Appraisal Tool « EMERGE ». Indian J Surg. 2020;82(5):930‑40.

38. Agrawal V, Yadav SK, Agarwal P, Sharma D. « EMERGE »: Construction of a simple quality appraisal tool for rapid review of laparoscopic surgery guidelines during COVID-19 pandemic. Br J Surg. 2020;107(11):e518‑9.

39. Wild C, Mayer-Ferbas J, Willemsen A. PP94 Pandemic Preparedness: EUnetHTA COVID-19 Rapid Response With « Rolling Collaborative Reviews (RCR) ». Int J Technol Assess Health Care. 2021;37(S1):18‑18.

40. Ballini L, Wild C, Djuric O, Mayer-Ferbas J, Willemsen A, Huic M. European Network for Health Technology Assessment’s Response to COVID-19: Rapid collaborative reviews on diagnostic tests and rolling and rapid collaborative reviews on therapeutics. Int J Technol Assess Health Care. 2022;38(1):e22.

41. Reynard C, Darbyshire D, Prager G, Jafar AJN, Naguib M, Oliver G, et al. Systematic literature search, review and dissemination methodology for the COVID-19 pandemic. BMJ Simul Technol Enhanc Learn. 2021;7(6):524.

42. Murad MH, Nayfeh T, Urtecho Suarez M, Seisa MO, Abd-Rabu R, Farah MHE, et al. A framework for evidence synthesis programs to respond to a pandemic. Mayo Clin Proc. 2020;95(7):1426‑9.

43. Munn Z, Twaddle S, Service D, Harrow E, Okwen PM, Schuenemann HJ, et al. Navigating evidence and guidance in a pandemic: Challenges, initiatives, and solutions for guideline developers. Scottish Charity NO SC 034407: Guidelines International Network; 2020.

44. Cochrane. Collaborating in response to COVID-19: editorial and methods initiatives across Cochrane. Cochrane Database Syst Rev. 2020;12(Suppl 1).

45. Scheinfeld L. LitCovid, iSearch COVID-19 portfolio, and COVID-19 Global literature on coronavirus disease. J Med Libr Assoc JMLA. 2022;110(2):279.

46. Chen Q, Allot A, Lu Z. LitCovid: An open database of COVID-19 literature. Nucleic Acids Res. 2021;49(D1):D1534‑40.

47. Juul S, Nielsen N, Bentzer P, Veroniki AA, Thabane L, Linder A, et al. Interventions for treatment of COVID-19: a protocol for a living systematic review with network meta-analysis including individual patient data (The LIVING Project). Syst Rev. 2020;9:1‑12.

48. Juul S, Nielsen EE, Feinberg J, Siddiqui F, Jørgensen CK, Barot E, et al. Interventions for treatment of COVID-19: of a living systematic review with meta-analyses and trial sequential analyses (The LIVING Project). PLoS One. 2021;16(3):e0248132.

49. Juul S, Nielsen EE, Feinberg J, Siddiqui F, Jørgensen CK, Barot E, et al. Interventions for treatment of COVID-19: A living systematic review with meta-analyses and trial sequential analyses (The LIVING Project). PLoS Med. 2020;17(9):e1003293.

50. Xin Y, Nevill CR, Nevill J, Gray E, Cooper NJ, Bradbury N, et al. Feasibility study for interactive reporting of network meta-analysis: experiences from the development of the MetaInsight COVID-19 app for stakeholder exploration, re-analysis and sensitivity analysis from living systematic reviews. BMC Med Res Methodol. 2022;22(1):26.

51. Evrenoglou T, Boutron I, Seitidis G, Ghosn L, Chaimani A. metaCOVID: A web‐application for living meta‐analyses of COVID‐19 trials. Res Synth Methods. 2023;14(3):479‑88.

52. Husson H, Howarth C, Neil-Sztramko S, Dobbins M. The National Collaborating Centre for Methods and Tools (NCCMT): Supporting evidence-informed decision-making in public health in Canada. Can Commun Dis Rep Releve Mal Transm Au Can. 2021;47(56):292‑6.

53. Dobbins M, Dubois A, Atkinson D, Bellefleur O, Betker C, Haworth-Brockman M, et al. Nimble, efficient and evolving: the rapid response of the National Collaborating Centres to COVID-19 in Canada. Health Promot Chronic Dis Prev Can Res Policy Pract. 2021;41(5):165‑70.

54. Neil-Sztramko SE, Belita E, Traynor RL, Clark E, Hagerman L, Dobbins M. Methods to support evidence-informed decision-making in the midst of COVID-19: creation and evolution of a rapid review service from the National Collaborating Centre for Methods and Tools. BMC Med Res Methodol. 27 oct 2021;21(1):231.

55. Kirkley MJ, Wright CJ. Rapid synthesis of a changing evidence base during the COVID-19 pandemic: the NeoCLEAR Project. J Perinatol. 2021;41(4):898‑900.

56. Southall S, Taske N, Power E, Desai M, Baillie N. Spotlight on COVID-19 rapid guidance: NICE’s experience of producing rapid guidelines during the pandemic. J Public Health Oxf Engl. 2021;43(1):e103‑6.

57. Fretheim A, Brurberg KG, Forland F. Rapid reviews for rapid decision-making during the coronavirus disease (COVID-19) pandemic, Norway, 2020. Eurosurveillance. 2020;25(19):2000687.

58. Zhou Q, Estill J, Wang Q, Wang Z, Shi Q, Zhang J, et al. Methodology and experiences of rapid advice guideline development for children with COVID-19: responding to the COVID-19 outbreak quickly and efficiently. BMC Med Res Methodol. 2022;22(1):89.

59. Thomas L, Jardine J, Relph S, Okano S, Shea M, Sadler M, et al. Developing high quality guidance at pace: the new « normal »? BJOG Int J Obstet Gynaecol. 2021;128(SUPPL 2):211.

60. Britt A, Yang E, Crittenden D, Bhangdia T, Nye B, Duffy EC, et al. Effects of COVID-19 treatments on cancer: A machine learning approach to synthesize clinical evidence at scale. Clin Cancer Res. 2021;27(6 SUPPL 1):P04.

61. Elmore R, Schmidt L, Lam J, Howard BE, Tandon A, Norman C, et al. Risk and Protective Factors in the COVID-19 Pandemic: A Rapid Evidence Map. Front Public Health. 2020;8:582205.

62. Weibel S, Popp M, Reis S, Skoetz N, Garner P, Sydenham E. Identifying and managing problematic trials: a Research Integrity Assessment (RIA) tool for randomized controlled trials in evidence synthesis. Res Synth Methods. 2023;14(3):357‑69.

63. Leong TD. Rapid evidence reviews to inform COVID-19 treatment guidelines in South Africa. J Public Health Afr. 2022;13(Supplement 1):62‑3.

64. Marshall AI, Archer R, Witthayapipopsakul W, Sirison K, Chotchoungchatchai S, Sriakkpokin P, et al. Developing a Thai national critical care allocation guideline during the COVID-19 pandemic: a rapid review and stakeholder consultation. Health Res Policy Syst. 2021;19(1):47.

65. Xu W, Zhang X, He Y, Dozier M, Owers B, Li X, et al. UNCOVER registry: A searchable online catalogue for COVID-19 evidence reviews. J Glob Health. 2020;10(2):020101.

66. Chater AM, Shorter GW, Swanson V, Kamal A, Epton T, Arden MA, et al. Template for rapid iterative consensus of experts (Trice). Int J Environ Res Public Health. 2021;18(19):10255.

# Supplementary file 3. Results of phase 3

The purpose of this phase was to identify ways to operationalize priorities identified in phase 1. A total of 26 persons participated in a deliberative workshop.

**Theme 1. Evidence ecosystem**

1. Setting up a crisis cell infrastructure in organizations
2. Implement information monitoring on a larger scale
3. Improve collaboration mechanisms within the health and social services system
4. Implement a provincial registry of requests to avoid duplication
5. Identify key people in organizations to receive requests
6. Recognize decision-support teams as essential
7. Mobilize more university researchers

**Theme 2. Knowledge translation**

1. Improving transparency in public communications
2. Quickly involve partners in the field for greater buy-in
3. Make evidence easily applicable and accessible
4. Gather rapid evidence products in one strategic location
5. Create spaces for citizens to share and value experiential knowledge

**Theme 3. Evidence synthesis methodology**

1. Adopt a proportional effort approach
2. Be transparent about methodological limitations and their impact
3. Give greater priority to the clarification of a rapid response request
4. Develop guidelines for translating data into recommendations
5. Give greater priority to teamwork to minimize bias
6. Train producers in strategic writing
7. Ensure better follow-up of rapid evidence synthesis

**Theme 4. Involvement of partners**

1. Involve partners quickly to keep them mobilized and responsive
2. Strengthen the health and social services system's capacity for involvement
3. Raise awareness of existing structures and available resources
4. Target projects requiring priority involvement of partners
5. Ensure fair remuneration without penalizing partners
6. Establish communication feedback mechanisms to ensure that the needs and knowledge of citizens are better taken into account
